# HAPLESS13-Mediated Trafficking of STRUBBELIG Is Critical for Ovule Development in Arabidopsis

**DOI:** 10.1371/journal.pgen.1006269

**Published:** 2016-08-19

**Authors:** Jia-Gang Wang, Chong Feng, Hai-Hong Liu, Fu-Rong Ge, Sha Li, Hong-Ju Li, Yan Zhang

**Affiliations:** 1 State Key Laboratory of Crop Biology, College of Life Sciences, Shandong Agricultural University, Tai’an, China; 2 State Key Laboratory of Molecular Developmental Biology, Institute of Genetics and Developmental Biology, Chinese Academy of Sciences, Beijing, China; UC Davis, UNITED STATES

## Abstract

Planar morphogenesis, a distinct feature of multicellular organisms, is crucial for the development of ovule, progenitor of seeds. Both receptor-like kinases (RLKs) such as STRUBBELIG (SUB) and auxin gradient mediated by PIN-FORMED1 (PIN1) play instructive roles in this process. Fine-tuned intercellular communications between different cell layers during ovule development demands dynamic membrane distribution of these cell-surface proteins, presumably through vesicle-mediated sorting. However, the way it’s achieved and the trafficking routes involved are obscure. We report that HAPLESS13 (HAP13)-mediated trafficking of SUB is critical for ovule development. *HAP13* encodes the μ subunit of adaptor protein 1 (AP1) that mediates protein sorting at the *trans*-Golgi network/early endosome (TGN/EE). The *HAP13* mutant, *hap13-1*, is defective in outer integument growth, resulting in exposed nucellus accompanied with impaired pollen tube guidance and reception. SUB is mis-targeted in *hap13-1*. However, unlike that of PIN2, the distribution of PIN1 is independent of *HAP13*. Genetic interference of exocytic trafficking at the TGN/EE by specifically downregulating *HAP13* phenocopied the defects of *hap13-1* in SUB targeting and ovule development, supporting a key role of sporophytically expressed SUB in instructing female gametogenesis.

## Author Summary

Ovules, being the progenitors of seeds, hold an important role in crop production. Ovule is a complex organ connecting two generations, i.e. diploid sporophytic cells and haploid female gametophytes (FG). Fertilization of female gametes leads to the development of embryo and endosperm while the sporophytic cells of ovules become seed coats. Because of its importance for plant reproduction, the development of ovules is tightly controlled. The integument cells are divided in a polar way so that integument cell layers fully enclose a mature FG, leaving an opening only for pollen tube entrance. The integuments also instruct FG development, synchronizing the two generations. Receptor-like kinases, especially STRUBBELIG (SUB), are crucial both for the establishment of ovular polarity and the intercellular communication between sporophytic and gametophytic cells. However, how SUB is post-translationally regulated, as would be expected for cell-surface proteins, is unclear. We report here that the dynamic trafficking of SUB via a subpopulation of endosomes is crucial for its functionality during ovule development. The membrane association of SUB was impaired when the recycling route from the *trans*-Golgi network/early endosome to the plasma membrane was disrupted in *hap13-1*, a mutant of *HAPLESS13*. We further demonstrate that defects of SUB targeting in the outer integuments compromised FG development, suggesting an intriguing intercellular communication. Our results highlight the importance of vesicular trafficking in the establishment of ovular polarity and in the communications between spatially separated cells.

## Introduction

Tissue or planar polarity that distinguishes multicellular organisms from unicellular ones requires coordinated cell behavior within a tissue or the plane of a tissue layer through intercellular communications and intracellular asymmetry [1,2]. Ovule development in angiosperms manifests the complexity by establishing and maintaining polarity in the proximal-distal axis, along which three morphologically distinct units are observed: the nucellus at the distal end harbors the megaspore mother cell (MMC) that undergoes meiosis to form an embryo sac, i.e. the female gametophyte (FG); from the chalaza at the central region, outer and inner integuments initiate growth, eventually enveloping the nucellus; the proximal funiculus connects the ovule to the placenta [1,3–6]. Fine-tuned intercellular communications are critical for primordium initiation, pattern formation, planar morphogenesis and female gametogenesis, collectively leading to ovule development [1,3,7].

Intercellular communication in plant development is mainly controlled by the phytohormone auxin as well as receptor-like kinases (RLKs) [2,8,9]. Auxin has been attributed as an instructive factor in the establishment and maintenance of tissue or planar polarity, through its asymmetric gradient [2,8,10,11]. Auxin gradient or maximum is a result of local biosynthesis, metabolism, and most importantly, transport facilitated by auxin influx and efflux carriers [8,10,11]. PIN-FORMED (PIN), the auxin efflux carriers, is encoded by 8 genes in Arabidopsis (*PIN1*-*PIN8*), whose dynamic distribution, often asymmetric, is essential for auxin gradient [8,10,11]. Among the eight PINs, PIN1 was reported to be critical for ovule development [8,12,13]. Tissue or planar polarity is also regulated by cellular perception and responses of neighboring cells through RLKs, whose extracellular domains enable signal perception while cytoplasmic domains allow intracellular signal relay [9]. Mutations at a few RLK-coding genes, such as *STRUBBELIG* (*SUB*), *ARABIDOPSIS CRINKLY4* (*ACR4*), and *ERECTA*-family genes (*ER* and *ERL*s), resulted in defective ovular development [14–16].

The membrane distribution of PINs and RLKs is dynamically regulated by vesicle trafficking-mediated protein sorting [11,17–19]. In the past decade, extensive studies revealed the key role of vesicle trafficking on the dynamic asymmetry of PINs [11,17,19]. RLKs are another dominant class of transmembrane (TM) proteins whose dynamic distribution through vesicles has been under close scrutiny [18]. The internalization of RLKs could be constitutive or induced whereas the internalized RLKs may recycle back to the PM non-selectively or selectively, as well as go to vacuoles for degradation [18]. As a consequence of their differential distributions, intracellular signaling is enhanced, reduced, or switched [18]. Therefore, vesicle trafficking-mediated protein sorting is fundamental to development. However, little is known on vesicle trafficking during ovule development, the trafficking routes involved, the key proteins vesicle-sorted, as well as the regulators of protein sorting.

An exocytic or anterograde trafficking route and an endocytic or retrograde trafficking route operate in plant cells to mediate protein sorting within the endomembrane system [20–22]. The two routes converge on the *trans*-Golgi network/early endosome (TGN/EE) [23], a likely heterogeneous group of endosomes equal to the combination of metazoan sorting endosomes, early endosomes, and recycling endosomes [21,22]. Protein sorting within the endomembrane system requires adaptor protein (AP) complexes, i.e. tetrameric protein complexes for cargo selection and coat recruitment [20,22,24]. Among the five AP complexes in eukaryotes, AP1 is associated with the TGN/EE and thus plays essential roles in protein sorting in plant cells [25–27]. AP1 consists of two large subunits (γ, β), one medium subunit (μ), and a small subunit (σ) [20,22,24]. We and others previously reported the identification of Arabidopsis *AP1μ*, *HAPLESS13* (*HAP13*)/*AP1MU2* [25–27]. Mutations of *HAP13* severely reduced male gametophytic transmission [25,27,28] whereas its homozygous mutants showed growth retardation [27]. Key TM proteins such as PIN2 [25,27], BRI1 [27], and KNOLLE [26] were mis-localized in the mutant of *HAP13*, *hap13-1*, demonstrating the key role of *HAP13* in TGN/EE-centered protein sorting.

We report here that HAP13-mediated protein sorting at the TGN/EE is critical for ovule development, specifically the asymmetric growth of outer integuments. By scanning electron microscopy (SEM), plastic section, and confocal laser scanning microscopy (CLSM), we show that *hap13-1* is defective in outer integument growth, leading to exposed or protruding nucellus. The defect in outer integuments severely affected female gametogenesis such that a large portion of *hap13-1* ovules did not contain a well-patterned embryo sac. Consequently, pollen tube guidance and reception were impaired, resulting in reduced female fertility. We further show that the key RLK for ovule development, SUB, was mis-targeted in *hap13-1*, correlating with the phenotypic similarity in ovules between *hap13-1* and *SUB* mutants. However, unlike PIN2 whose membrane distribution relies on *HAP13* [25,27], the asymmetric targeting of PIN1 at the nucellus was intact in *hap13-1*, suggesting distinct sorting mechanisms for different PINs. Downregulating *HAP13* expression by RNA interference (RNAi) specifically in outer integuments phenocopied the ovular defects in *hap13-1*. Results presented here demonstrate the importance of TGN/EE-centered protein sorting in outer integument growth and provide clues for a deep understanding of molecular mechanisms underlying the establishment and maintenance of ovule polarity.

## Results

### Expression of *HAP13* during ovule development

**Fig 1:**
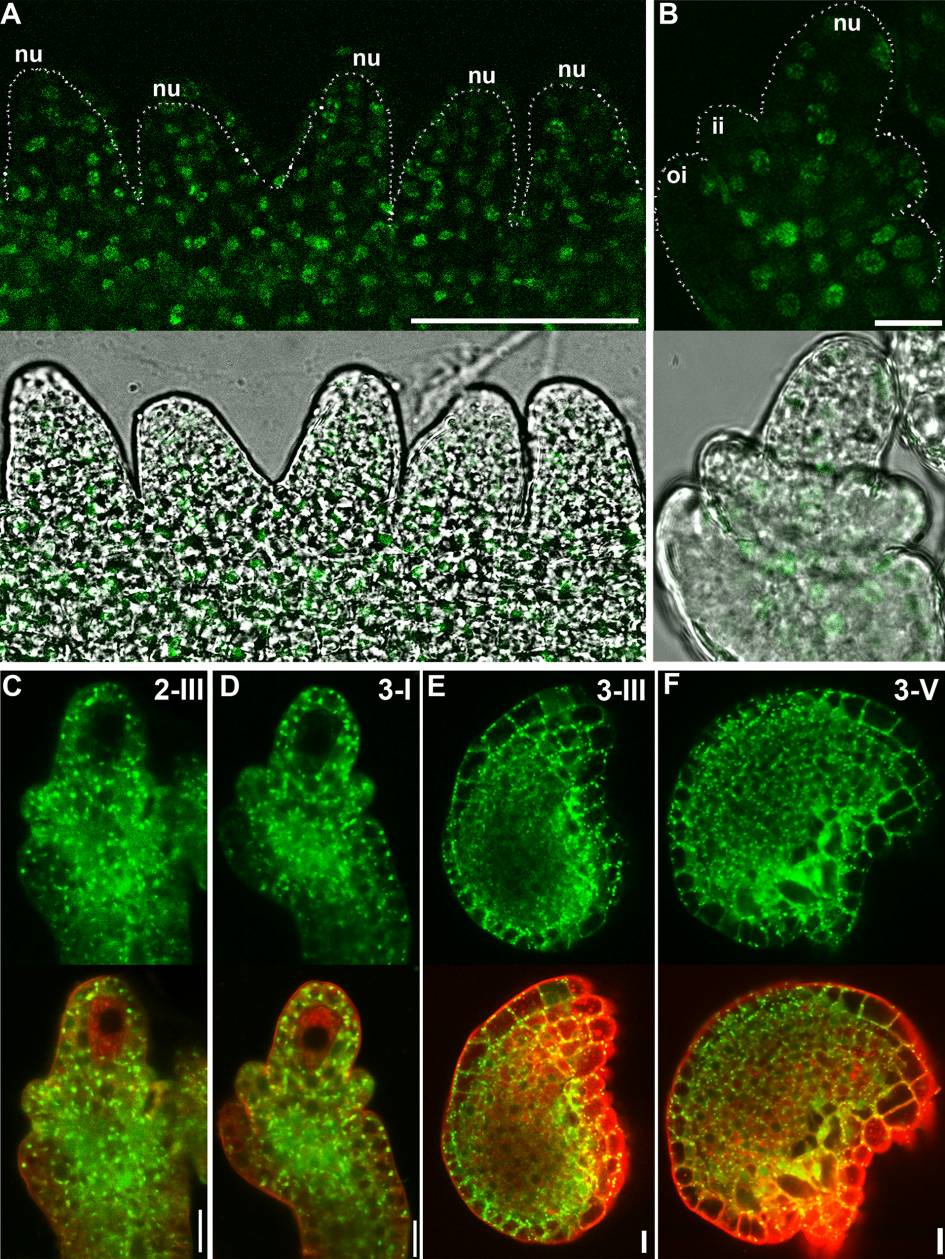
Expression of *HAP13* during ovule development. (A-B) Confocal laser scanning micrographs (CLSM) of *Pro*_*HAP13*_:NLS-YFP ovules at stage 2-I (A) or at stage 2-III (B). Silhouettes of developing ovular primordia (A) or ovule (B) were indicated with dotted lines. Nu nucellus; ii, inner integument; oi, outer integument. (C-F) CLSM of developing ovules from *HAP13g-GFP hap13-1* transgenic plants at stage 2-III (C), 3-I (D), 3-III (E), and 3-V (F). Merge images of the GFP channel and the RFP channel (Lysotracker red, to show cell silhouettes) are at the bottom of corresponding GFP channel images. Bars = 50 μm for (A); 10 μm for (B-F).

*HAP13* was expressed in various tissues and developmental stages [27]. To determine whether *HAP13* was expressed during ovule development, we generated a nuclear-localized YFP reporter line for *HAP13* (*Pro*_*HAP13*_:NLS-YFP). Confocal laser scanning microscopy (CLSM) revealed that *HAP13* was expressed in all cell layers during ovule development (Fig 1A and 1B). Finally, the expression of *HAP13* in ovules during development was confirmed by examining the *HAP13g-GFP hap13-1* transgenic plants (Fig 1C–1F). The expression of *HAP13* in ovules from primordium formation till maturation hinted at the possibility of its function in ovule development.

### Ovule developmental defects in *hap13-1*

**Fig 2:**
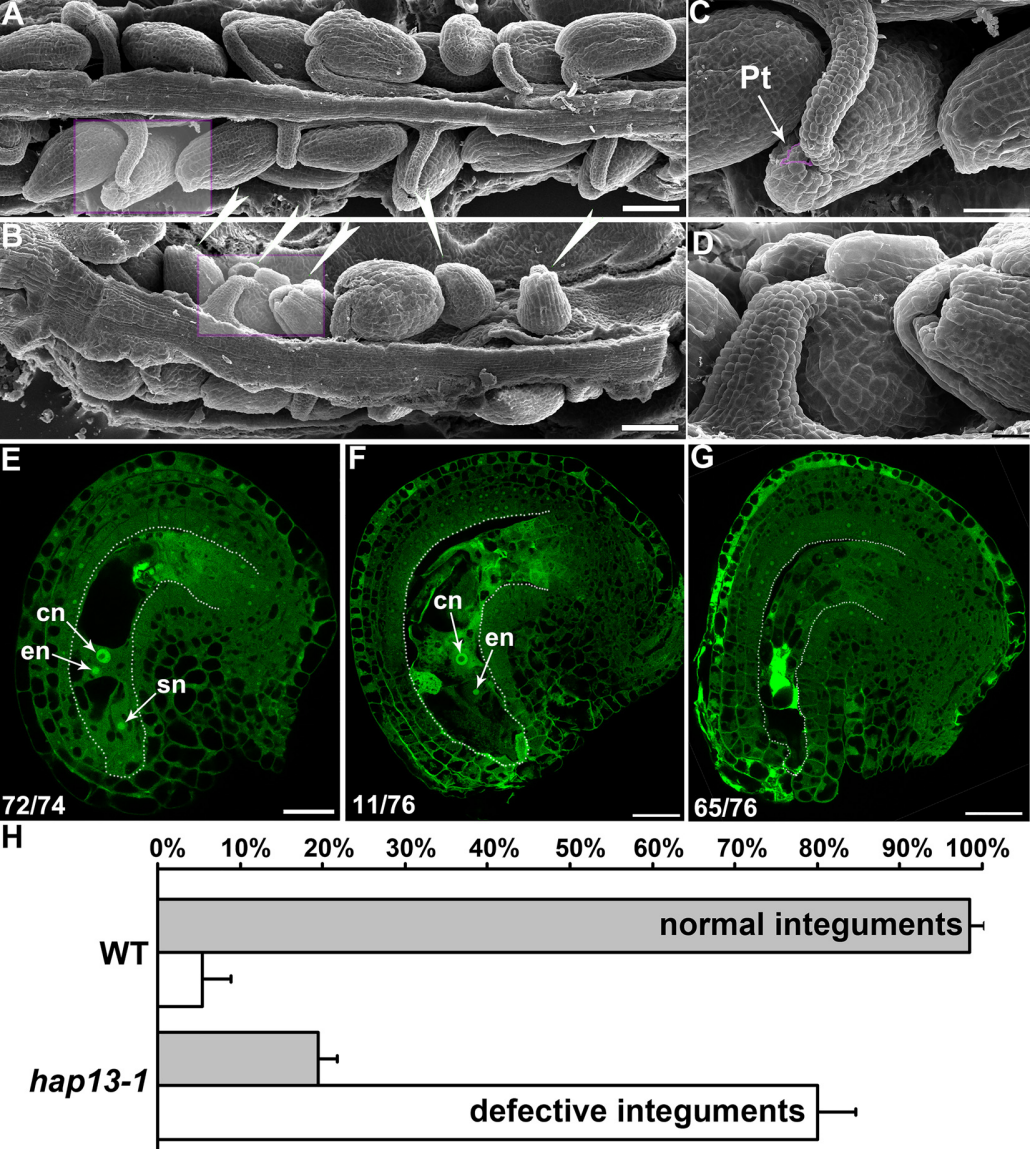
Ovule development in wild type and *hap13-1*. (A-B) Scanning electron micrographs (SEMs) of wild-type (A) or *hap13-1* (B) mature pistils at 48 hours after pollination (HAP). The defective *hap13-1* ovules are highlighted with arrowheads. (C-D) Close-ups of wild-type (C) or *hap13-1* (D) ovules as indicated in the shaded rectangle box in (A-B). An incoming pollen tube (Pt) at the micropyle of a wild-type ovule is false-colored pink. Two ovules with broken or incomplete outer integuments are shown in (D). (E-G) CLSM of a mature ovule from wild type (E), or from a mildly affected (F) or severely affected (G) *hap13-1* mutant. Mid-optical sections of stage 3-V ovules are shown. The number of displayed type among all ovules examined is shown at the bottom left. (H) Quantitative analyses of integument development. Results in (H) are means ± standard errors (SE, N = 15). Bars = 100 μm for (A-B); 50 μm for (C); 20 μm for (D); 25 μm for (E-G).

To determine whether *HAP13* was required for ovule development, we examined *hap13-1* pistils by SEM and CLSM analyses. Ovules are connected with the gynoecium through the funiculus (Fig 2A and 2B). At the distal end of the funiculus is a cleft, i.e. micropyle, formed by inner and outer integument cells enveloping the seven-celled embryo sac in wild type (Fig 2A–2D). In comparison, around 80% of *hap13-1* ovules (Fig 2H) were abnormal such that the surface cell layers were broken and thus only partially wrapping the inner structure (Fig 2B and 2D). At maturity, wild-type ovules contained well-patterned FGs with a central cell, an egg cell and two synergid cells (Fig 2E). By contrast, FGs were abnormal in over 80% of *hap13-1* ovules (Fig 2F and 2G). In addition, due to incomplete growth of outer integuments, embryo sacs in *hap13-1* ovules were exposed or protruding (Fig 2B and 2D).

### Sporophytically controlled female gametophytic function was compromised in *hap13-1*

**Fig 3:**
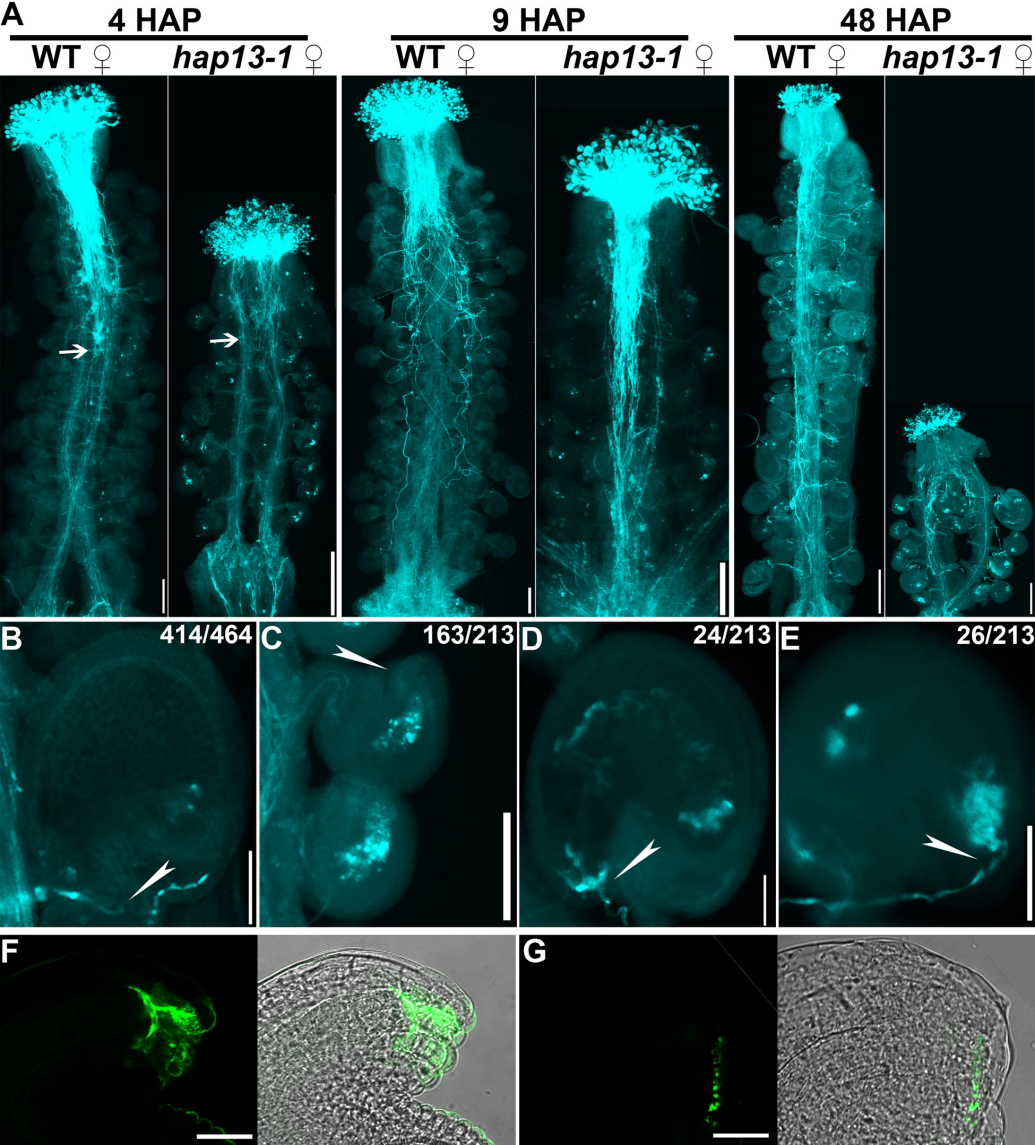
Pollen tube guidance and reception are compromised in the homozygous *hap13-1*. (A) Aniline blue staining of wild-type or *hap13-1* pistils emasculated and hand-pollinated with wild-type pollen at 4, 9, or 48 hours after pollination (HAP). Arrows indicate the growth front of pollen tubes. (B-E) Close-ups of a wild-type ovule (B) and *hap13-1* ovules of different categories, including *hap13-1* ovules unable to attract pollen tubes (C), those pollinated and fertilized (D), or those with overgrown pollen tubes (E) at 48 HAP. Frequency of displayed ovules in total ovules examined is shown on top. Arrowheads point at the micropylar region. (F-G) Immunofluorescence staining of a mature wild-type (F) or *hap13-1* ovule (G) using an anti-LURE antibody. Bars = 100 μm for 4 HAP images in (A), 200 μm for 9 HAP or 48 HAP in (A), 50 μm for (B-E), 20 μm for (F-G).

Pollination of emasculated *hap13-1* pistils with wild-type pollen only yields a few seeds although female transmission of *hap13-1* was normal [27]. To determine the cause of the reduced female fertility, we performed aniline blue staining of emasculated wild-type or *hap13-1* pistils hand-pollinated with wild-type pollen at different time points. At 4 hours after pollination (HAP), pollen tubes already entered the transmitting track in wild-type pistils but only exited the style in *hap13-1* pistils (Fig 3A). It suggested that *hap13-1* pistils were compromised in the support of pollen tube growth. At 9 HAP, pollen tubes reached the bottom of both wild-type and *hap13-1* pistils (Fig 3A). However, *hap13-1* pistils were much shorter than those of wild type due to a reduced number of ovules (Fig 3A). Therefore, pollen tube growth inside *hap13-1* pistils was substantially slow. At 48 HAP when wild-type pistils contained enlarged ovules indicative of successful fertilization (Fig 3A and 3B), only few ovules were enlarged in *hap13-1* pistils (Fig 3A and 3D). Most *hap13-1* ovules were not targeted by pollen tubes (163 out of 213, Fig 3C), indicating compromised pollen tube guidance. Another portion of *hap13-1* ovules did attract pollen tubes (26 out of 213, Fig 3E). However, these ovules failed to instruct the cessation of pollen tube growth, resulting in overgrown pollen tubes inside embryo sacs and failed fertilization (Fig 3E).

To determine the reason of reduced pollen tube guidance in *hap13-1*, we performed immunofluorescence staining on wild-type and *hap13-1* ovules using an anti-LURE antibody [29]. Because LURE is a key peptide secreted by synergid cells to attract pollen tubes [30,31], we wanted to test whether *hap13-1* ovules were compromised in the production of LURE. In mature wild-type ovules, strong signals were detected in the micropylar region (Fig 3F), indicative of LURE secretion. By contrast, *hap13-1* ovules (45 out of 57 examined) showed a substantial reduction of fluorescence signals (Fig 3G). This result explained the reduced pollen tube guidance of *hap13-1* ovules. Next, we introduced an egg cell-specific reporter *Pro*_*DD45*_:GUS [32] into *hap13-1*. By histochemical analysis of the homozygous transgene *Pro*_*DD45*_:GUS in the heterozygous and homozygous *hap13-1*, we found that egg cells were present in almost all ovules of the heterozygous *hap13-1* pistils, comparable to those of wild type (S1 Fig). However, only a few ovules of the homozygous *hap13-1* pistils showed GUS signals (S1 Fig). These results demonstrated that defective patterning of *hap13-1* FGs was caused by defects in surrounding sporophytic tissues.

### Planar polarity during ovule development was compromised in *hap13-1*

**Fig 4:**
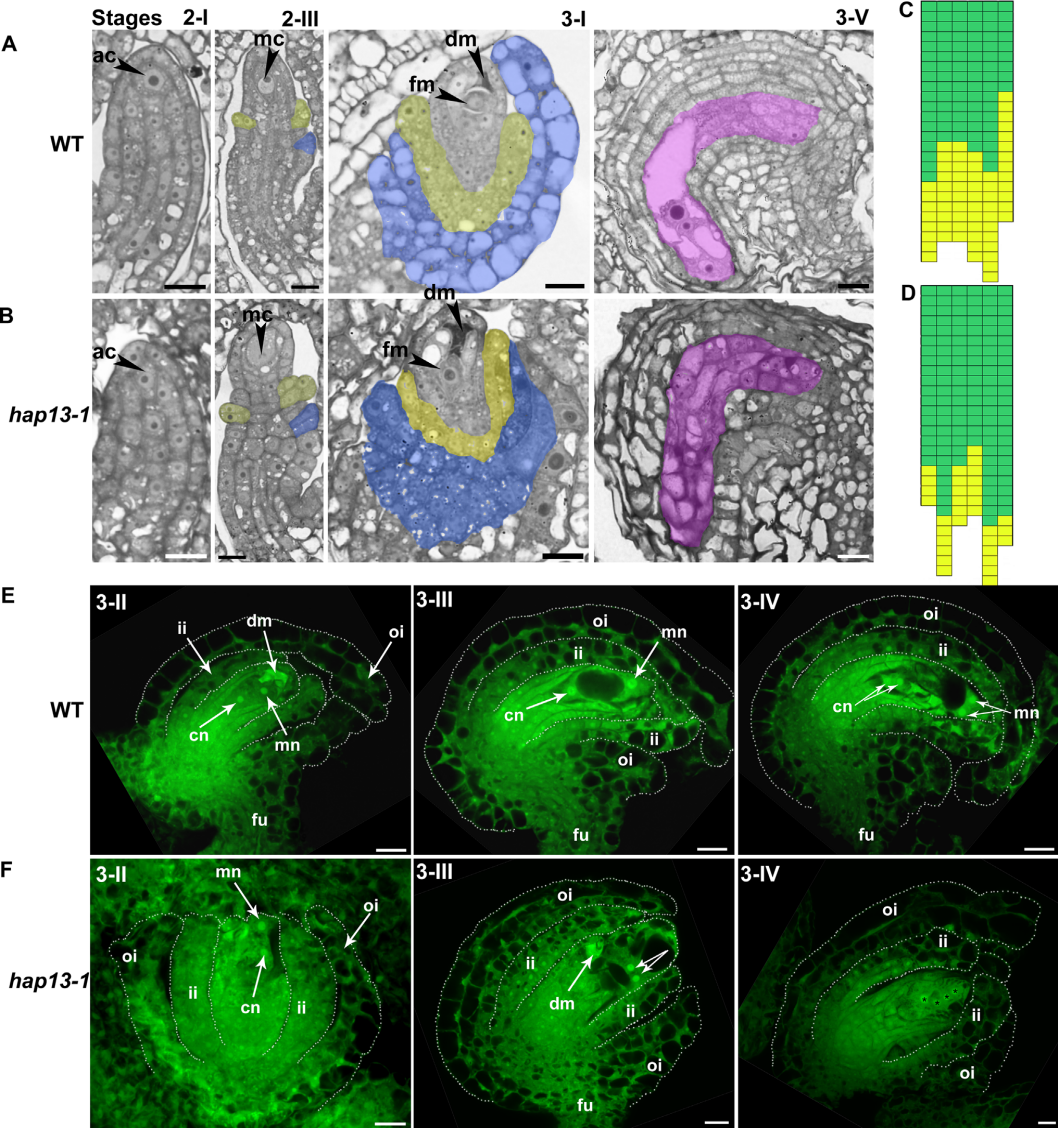
Planar polarity during ovule integument growth is compromised in *hap13-1*. (A-B) Semi-thin sections of wild-type (A) or *hap13-1* ovules (B) at developmental stage 2-I, 2-III, 3-I, or 3-V. ac, archesporial cell; dm, degenerating megaspore; fm, functional megaspore. Yellow-shaded areas are cells of inner integuments. Blue-shaded areas are cells of outer integuments. Pink-shaded areas indicate the embryo sacs. (C-D) Cell number and size of wild-type (C) or *hap13-1* (D) outer integuments. Each column represents one stage 3-V ovule; the number of cubes in each column indicates the number of countable outer integument cells in a stage 3-V ovule; green cubes indicate small cells (area between 0.2 and 0.4, a.u.) while yellow cubes indicate large cells (are between 0.4 and 1, a.u.). (E-F) CLSMs of wild-type (E) or *hap13-1* ovules (F) at stage 3-II, 3-III, or 3-IV. Ovules were stained with PI and mid-optical sections were shown. Because some *hap13-1* ovules were completely avoid of embryo sac patterning and thus impossible to determine developmental stages, only *hap13-1* ovules with visible nuclei are shown in (F). cn, chalazal nucleus; fu, funiculus; ii, inner integument; oi, outer integument; mn, micropylar nucleus. Bars = 10 μm.

To determine at which stage *hap13-1* was defective during ovule development, we performed semi-thin plastic sections and CLSM on ovules at different developmental stages. The phenotype of *hap13-1* in ovule development was variable, ranging from severely affected to normal and fertile ovules. Seventy percent of ovules from *hap13-1* mutants displayed developmental irregularities (N = 256 of *hap13-1* ovules for percentage calculation). Developmental defects of *hap13-1* started at stage 2-III (Fig 4B), immediately after outer integuments were initiated [5,6]. Upon initiation at an abaxial position, outer integuments in *hap13-1* sometimes failed to spread around the circumference of ovules, resulting in incomplete outer integuments (Fig 4B and 4F), unlike those in wild type where outer integuments fully covered inner integuments (Fig 4A and 4E). Cell expansion in outer integuments of *hap13-1* was impaired (Fig 4C and 4D). This irregular cell growth and possibly division in *hap13-1* resulted in ovules with exposed inner integuments, enveloped by fragmented outer integuments (Fig 4B and 4F). The two-cell-layered organization of outer integuments was also disrupted (Fig 4B and 4F), likely caused by mis-oriented division planes (Fig 4B and 4F). About 80% ovules in *hap13-1* contained abnormal FGs, which always correlated with abnormal growth of outer integuments (Fig 4F).

### SUB is mis-targeted during ovule development in *hap13-1*

The phenotypic defects of *hap13-1* ovules resembled that of *sub* mutants to a great extent [14] and an earlier study showed that SUB could be internalized upon treating root cells with Brefeldin A (BFA) [33], a fungal toxin that inhibits post-Golgi secretion [34]. We thus hypothesized that membrane distribution of SUB relied on HAP13-mediated protein sorting at the TGN/EE. To test this hypothesis, we analyzed the distribution of SUB in *hap13-1* by introducing a YFP-translational fusion of *SUB* genomic fragment driven by its endogenous promoter, SUBg-YFP [35]. A post-transcriptional regulation was proposed earlier based on the differential distribution patterns of a full *SUB* genomic fusion and a fusion with the coding sequence of *SUB* [33,36,37]. Nevertheless, both were able to fully restore ovular defects of *sub* mutants [33,36,37].

**Fig 5:**
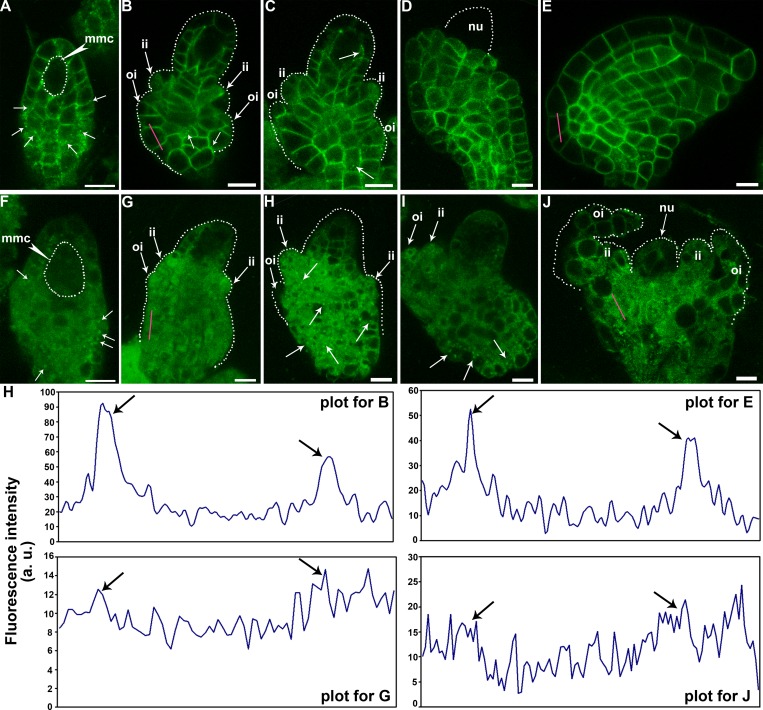
Dynamic localization of SUB is disrupted in *hap13-1* ovules. (A-E) Representative CLSM of ovules at different developmental stages from the SUBg-YFP transgenic plants. Dotted lines illustrate the shape of either megaspore mother cell (mmc), nucellus (nu), or of developing ovules (B-C). Arrows point at inner integuments (ii), outer integuments (oi), or punctate vesicles (without labeling). Note that SUBg-YFP was asymmetrically distributed in epidermal cell layers, i.e. excluded from the outward plasma membrane in (B-C). (F-J) Representative CLSM of ovules at different developmental stages from the SUBg-YFP;*hap13-1* transgenic plants. Dotted lines illustrate the shape of either megaspore mother cell (mmc) or of developing ovules (G-H), and (J). Arrows point at inner integuments (ii), outer integuments (oi), or punctate vesicles (without labeling). Note that SUBg-YFP was more randomly distributed intracellularly and in larger vesicles. Four independent experiments involving 40 ovules at various developmental stages showed similar results. (H) Fluorescence intensity plots along the red lines across a typical outer integument cell in (B), (E), (G), and (J) are shown. The y-axis values represent signal intensity. The arrows indicate the signal accumulation at the plasma membrane. Bars = 10 μm.

In ovules of the SUBg-YFP transgenic lines, YFP was barely detectable in the nucellus but present at both inner and outer integuments during early stages of ovule development as well as in all cell types in mature ovules (Fig 5A–5E), as reported [33,36,37]. Immediately after ovular primordia formation, YFP was distributed evenly at the plasma membrane (PM) of epidermal cell layer and also in a few vesicles (Fig 5A). With the development of ovules, YFP signals became asymmetric, i.e. excluded from the outward PM of the epidermal cell layer (Fig 5B and 5C). Punctate vesicles were still detectable (Fig 5B–5D). At later stages when outer integuments began rapid asymmetric elongation and finally wrapped embryo sacs, punctate vesicles labeled by YFP could hardly be detected (Fig 5E). Also YFP was more evenly distributed at the PM of the epidermal cell layer (Fig 5D and 5E). By contrast, YFP signals were mostly diffused in the cytoplasm rather than at the PM of *hap13-1* ovules (Fig 5F–5J). Punctate vesicles, much larger than those in wild type, were visible despite the cytosolic signals (Fig 5F–5I). The asymmetry of YFP signals observed in wild type was often compromised such that it labeled the outward PM of the epidermal cell layer (Fig 5I). These results indicated that SUB is dynamically trafficked during ovule development and this process relies on *HAP13*. Interestingly, YFP signals were substantially reduced in *hap13-1* despite that the same transgene was analyzed (Figs 5H and S2). Transcript analysis confirmed that the mRNA abundance of *SUB* was comparable between wild type and *hap13-1* (S2 Fig), suggesting a post-transcriptional regulation of *SUB*. Indeed, a post-translational regulation was proposed earlier because treatment of the 26S proteasome inhibitor MG132 reduced the protein level of SUB [33].

To further verify that the subcellular targeting of SUB depends on *HAP13*, we analyzed SUB dynamic targeting in roots by using the fluorescence lipophilic dye FM4-64 together with pharmacological treatments. FM4-64 enters cells via endocytosis, labeling various endomembrane compartments over time and finally reaching vacuolar membrane, i.e. the tonoplast [38]. As shown in ovules, SUB was localized at the PM of wild-type root cells, overlapping with FM4-64 immediately after pulse labeling (S3 Fig). Treatment of roots with BFA at the presence of cycloheximide (CHX) resulted in the accumulation of SUB signals into BFA compartments co-labeled by FM4-64 (S3 Fig). Because BFA compartments formed at the presence of CHX contain only proteins internalized from the PM [27], the result indicated that SUB was constitutively internalized from the PM. The internalization of SUB upon BFA treatment was comparable between wild type and *hap13-1* (S3 Fig), which is consistent with the intact endocytosis in *hap13-1* [27]. Washout of BFA with CHX caused complete re-distribution of SUB from the BFA compartments to the PM in wild type, when FM4-64 labeled the tonoplast via vacuolar trafficking (S3 Fig). By contrast, the internalized SUB was insensitive to BFA washout in *hap13-1* (S3 Fig). Instead, it remained sequestered at the BFA compartments together with FM4-64 (S3 Fig). These results suggested that the recycling of SUB from the TGN/EE to the PM requires *HAP13*.

### PIN1 localization and auxin responses during ovule development in *hap13-1*

**Fig 6:**
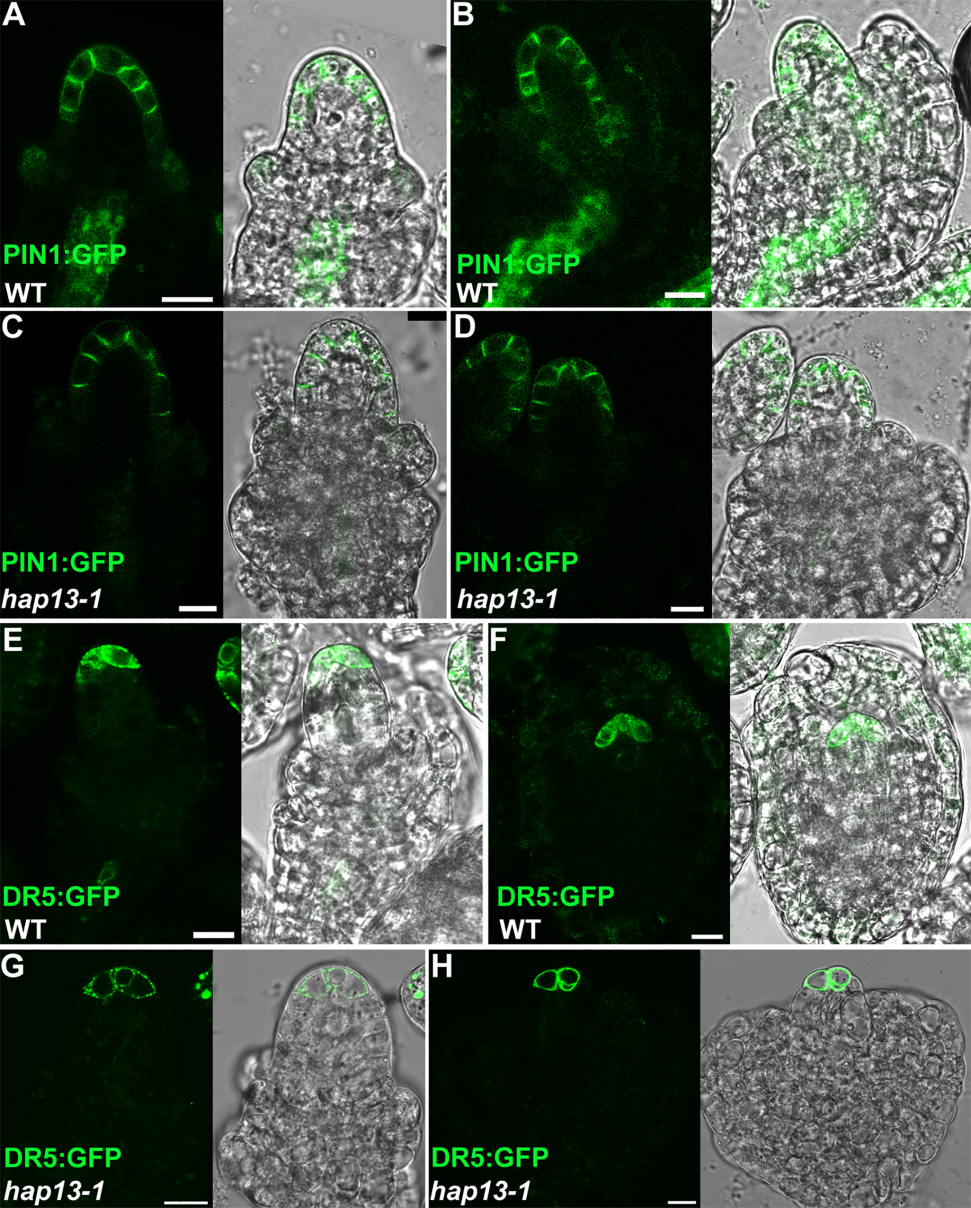
Auxin maximum and polar localization of PIN1 in *hap13-1* ovules. (A-D) PIN1::GFP in wild-type (A, B) or in *hap13-1* (C, D) ovules at stage 2-II (A, C) or at stage 3-I (B, D) by CLSM. (E-H) DR5:GFP in wild-type (E, F) or in *hap13-1* (G, H) ovules at stage 2-III (E, G) or at stage 3-II (F, H) by CLSM. Merges of bright-field and fluorescent images are placed at the right of corresponding fluorescent images. Bars = 10 μm.

Except for SUB, PIN1 is another key component for ovule development by channeling auxin flux to the primordia tip [8,13]. We and others previously showed that the dynamic targeting of PIN2 relies on *HAP13* whose mutations resulted in defective gravitropism due to mis-targeting of PIN2 [25,27]. In addition, both PIN1 and PIN2 belong to the same subgroup of PINs with a long hydrophilic loop [39]. We thus hypothesized that PIN1 dynamic targeting also relied on *HAP13*. To test this hypothesis, we introduced the *PIN1* genomic-GFP translation fusion, PIN1::GFP [8], into *hap13-1* and followed its membrane distribution at different developmental stages in ovules. PIN1 was detected at the outer cell layer of the nucellus, pointing toward the distal tip (Fig 6A and 6B). Surprisingly, the asymmetric membrane localization of PIN1 was comparable between wild type and *hap13-1* despite the abnormal integument growth of *hap13-1* (Fig 6C and 6D). Consistent with the intact PIN1 distribution in the nucellus, auxin responses at the nucellus (Fig 6E and 6F) as indicated by the activity of DR5:GFP [40] also did not show abnormality in *hap13-1* (Fig 6G and 6H).

To determine whether the *HAP13*-independent PIN1 targeting also applied to other cells, we examined the localization of PIN1 in the roots of *hap13-1*. No substantial difference of PIN1 distribution was found between wild type and *hap13-1* in root cells (S4 Fig), confirming the *HAP13*-independency of PIN1 targeting.

### Genetic interference of *HAP13* in outer integuments mimics *hap13-1* in ovule development

We previously showed that a full length *HAP13* genomic fragment fully restored developmental and cellular defects in *hap13-1* [27], confirming the mutant identity of *hap13-1*. Indeed, HAP13g-GFP completely restored ovule development of *hap13-1* (Fig 1C–1F). However, *HAP13* is constitutively expressed and its mutations results in a full spectrum of developmental defects [27]. Thus, the defects in outer integument growth of *hap13-1* might be non-cell-autonomous. To exclude the possibility, we used an RNAi approach. We generated *Pro*_*UBQ10*_:HAP13-RNAi and analyzed the transcript level of *HAP13* in seedlings as well as examined the effect of HAP13-RNAi on vesicle trafficking. Six out of twenty *Pro*_*UBQ10*_:HAP13-RNAi transgenic lines were randomly selected. Real-time quantitative PCRs (qPCRs) verified a significant reduction of *HAP13* transcripts by *Pro*_*UBQ10*_:HAP13-RNAi (S5 Fig). In addition, *hap13-1* was insensitive to BFA washout [27], which was confirmed in root epidermal cells of *Pro*_*UBQ10*_:HAP13-RNAi transgenic lines by an uptake experiment using the lipophilic dye FM4-64 (S5 Fig). These results verified the efficient down-regulation of *HAP13* by RNAi.

To downregulate the expression of *HAP13* specifically in outer integuments, we generated a construct containing *HAP13*-RNAi driven by the promoter of *INNER NO OUTER* (*Pro*_*INO*_). *Pro*_*INO*_ was reported previously for its specific expression in ovules based on RNA *in situ* hybridization [41]. To test whether *Pro*_*INO*_ was specific for outer integuments where severe growth defects were observed in *hap13-1*, we performed histochemical GUS staining on *Pro*_*INO*_:GUS transgenic ovules at different stages. GUS signals were first observed at the stage 2-I in cells to be differentiated into outer integuments (S6 Fig). The asymmetrically distributed GUS signals, correlating with the asymmetric growth of outer integuments, were getting stronger during development. GUS signals were nearly undetectable when embryo sacs were fully formed (S6 Fig). Although *Pro*_*INO*_ showed a slightly wider spatial activity than the transcripts of *INO* based on *in situ* hybridization [42], the histochemical GUS analysis nevertheless demonstrated that *Pro*_*INO*_ was able to driven gene expression specific in outer integuments during ovule development.

**Fig 7:**
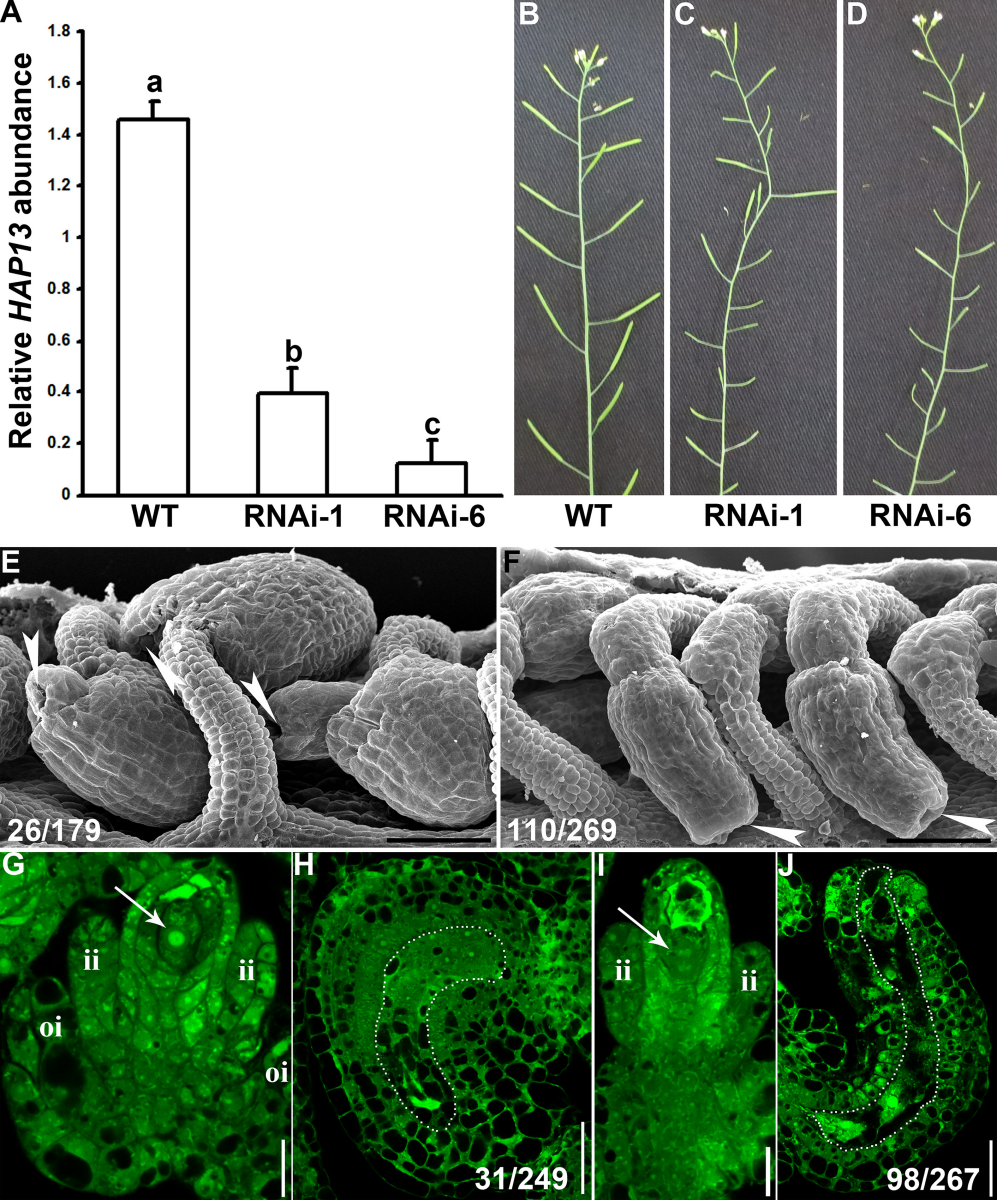
Genetic interference of *HAP13* phenocopies *hap13-1* in ovule development. (A) Relative transcript abundance of *HAP13* in wild type or two *Pro*_*INO*_:HAP13-RNAi transgenic lines (RNAi-1 and RNAi-6) by qPCRs. RNAs were extracted from maturing ovules at flower stage 11–12. Results shown are means ± SE (N = 3). Means with different letters are significantly different (One way ANOVA, Tukey-Kramer method, P<0.05). (B-D) Representative inflorescence from wild type (B) or two *Pro*_*INO*_:HAP13-RNAi transgenic lines (C-D). Note numerous non-elongating siliques at the inflorescence of the *Pro*_*INO*_:HAP13-RNAi transgenic lines. (E-F) SEMs of developing ovules from RNAi-1 (E) or RNAi-6 (F) transgenic line. Arrowheads point at the micropylar region. The number of displayed type among all ovules examined is shown at the bottom left. (G-J) Optical sections of developing ovules from RNAi-1 (G, H) or RNAi-6 (I, J) *Pro*_*INO*_:HAP13-RNAi transgenic line at stage 3-I (G, I) or stage 3-V (H, J). Arrows indicate functional megaspore (FM). es, embryo sac; ii, inner integument; oi, outer integument. Dotted lines highlight the embryo sacs. The number of displayed type among all ovules examined is shown at the bottom left. Bars = 50 μm for (E-F); 10 μm for (G), (I); 25 μm for (H), (J).

Over 20 independent *Pro*_*INO*_:HAP13-RNAi transgenic lines were generated, all of which showed reduced fertility to different extents as compared to wild type (Fig 7B). Two lines with strong or medium defects of ovule development were analyzed by qPCRs using maturing ovules as the materials (floral stage 11–12) (Fig 7A). These two *Pro*_*INO*_:HAP13-RNAi lines representing medium or strong reduction of *HAP13* expression (RNAi-1 and RNAi-6) were used for further analysis by CLSM and SEM. SEM of developing ovules revealed that HAP13-RNAi resulted in severe reduction of outer integument growth (Fig 7E–7F), similar to and sometimes severer than that in *hap13-1*, which is a weak rather than null allele of *HAP13* [27,28]. At stage 3-I, the mild knockdown line showed regular two-cell-layer inner integuments symmetrically surrounding the nucellus (Fig 7G). By contrast, the growth of outer integuments, although initiated, was severely reduced (Fig 7G). For the severe knockdown line, the growth of outer integuments was arrested immediately after initiation, i.e. the enlarged cells of initiating outer integuments failed to proliferate or grow (Fig 7I). As a result, ovules at maturation stage resembled stubby tubules with only inner integuments enveloping the nucellus (Fig 7J). Downregulating *HAP13* specifically in outer integuments phenocopied *hap13-1*, indicating that defects in ovule development in *hap13-1* were not a secondary effect of *HAP13* through constitutive expression elsewhere.

Specifically downregulating *HAP13* in outer integuments resulted in defects of embryo sac patterning such that most ovules of the *Pro*_*INO*_:HAP13-RNAi transgenic plants contained extruding nucellus without the embryo sac at maturation (Fig 7), similar to that shown in *hap13-1* (Fig 4). However, it was also possible that genetic interference of *HAP13* affected ovule development and thus altered the expression specificity of *Pro*_*INO*_, leading to the defects in embryo sac patterning. To exclude the possibility, we generated a binary vector containing two independent expression cassettes, *Pro*_*INO*_:HAP13-RNAi and *Pro*_*INO*_:NLS-YFP. We generated transgenic plants and applied CLSM to verify the spatial specificity of *Pro*_*INO*_ in the transgenic lines. In severely affected lines where outer integuments failed to extend, hardly any NLS-YFP signals could be observed in either cell layers within the ovules (S7 Fig). In mildly affected lines where outer integuments showed incomplete extension and wrapped inner integuments partially, NLS-YFP signals were specifically present in outer integuments (S7 Fig). These results confirmed the specific expression of *Pro*_*INO*_ even when the growth of outer integuments was compromised by downregulating *HAP13*.

### DISCUSSIONS

We show here that the *hap13-1* mutant was compromised in the asymmetric growth of outer integuments, as a result of both division and elongation defects (Figs 2 and 4). Although *HAP13* is expressed in all cells during ovule development (Fig 1), its function does not seem to be crucial for inner integuments because transgenic plants expressing HAP13-RNAi in outer integuments mimicked the *hap13-1* mutant (Fig 7). Despite the fact that *hap13-1* contained a large portion of ovules with disrupted FGs (Fig 2) and was compromised in pollen tube guidance and reception (Fig 3), *HAP13* is also not crucial for female gametogenesis because the heterozygous *hap13-1* plants has normal female gametogenesis and female transmission [27] comparable to those in wild type (S1 Fig).

As a subunit of the AP1 complex, HAP13 is critical for vesicle-mediated protein sorting from the TGN/EE to the PM and to vacuoles [25,27]. SUB is the most extensively studied RLK regulating ovule development [14,33,37], in addition to its roles in epidermal patterning and cell fate determination in roots [36,43]. It was reported that SUB accumulates into BFA compartments [33]. However, it was unclear whether SUB within the BFA compartments was trapped during its secretion to the PM or from the PM through constitutive recycling, like ACR4 [44], another RLK regulating epidermal patterning [15]. By using BFA treatment at the presence of CHX, we demonstrated that SUB is constitutively internalized from the PM (S3 Fig). BFA washout resulted in the disappearance of SUB from the BFA compartments in wild type but not in *hap13-1* (S3 Fig), suggesting that SUB recycles from the TGN/EE to the PM and this process depends on *HAP13*. The PM association of SUB was substantially affected in *hap13-1* ovules (Fig 5), suggesting that HAP13-mediated SUB recycling plays a role in its PM association in ovules. Intriguingly, in root epidermal cells, no substantial difference was observed on the PM association of SUB between wild type and *hap13-1* (S3 Fig), implying that the recycling pathway might be cell- or tissue-specific.

Although it is tempting to propose that HAP13-mediated sorting of SUB at the TGN/EE is critical for pattern formation during ovule development, we can not exclude the possibility that the mis-targeting of other transmembrane proteins also contributes to the observed ovule defects of *hap13-1*. SUB is the best studied and likely most important RLK regulating integument growth [14]. However, other RLKs such as ACR4 and ER family members are also involved in this process [15,16]. ACR4 was present both at the PM and at endosomes via vesicle trafficking [44] and thus is likely sorted via AP1. Whether ER and ERLs are dynamically trafficked via vesicles is currently unknown. However, the *hap13-1* mutant showed compact inflorescence architecture as contrast to that of the ecotype Ws in which the mutant was generated [27]. Because mutations at *ER* and *ER* family members impaired cell proliferation in aerial organs and led to similar inflorescence architecture [45], it implies HAP13-dependency of *ER* function. Indeed, *hap13-1* showed abnormal cell division during ovule development (Fig 4), which was less severe in the *sub* mutants [14]. A key protein involved in cytokinesis, KNOLLE, was reported to rely on *HAP13* for its dynamic targeting during cell division [26]. Similar regulators during ovule development may rely on *HAP13* for cell division to be properly executed.

Because PIN2 was mis-targeted in *hap13-1* [25,27], while PIN1 is another long-hydrophilic looped PIN similar to PIN2 [39], it turned out to be a surprise that the asymmetric localization of PIN1 is not affected in *hap13-1*, either during ovule development or in roots (Figs 6 and S4). Consistently, auxin response, which plays an instructive role in ovule polarity [8,13], is not compromised in *hap13-1* ovules (Fig 6), unlike the case in roots during gravitropic response [27]. The differential requirement of *HAP13* for the dynamic targeting of PIN1 and PIN2 suggested distinct trafficking routes involved. Indeed, a previous report showed that Endosidin1, a chemical inducing endocytosis, defines a compartment involved in the endocytosis of PIN2 but not that of PIN1 [19]. The differential requirement of PIN1 and PIN2 on *HAP13* indicates that the recycling of PIN1 and PIN2 to the PM also adopts different routes, probably through distinct regulators.

Another interesting point to note is sporophytic control of female gametogenesis. The defective pollen tube guidance and reception shown by *hap13-1* suggested a compromised female gametophytic function [46,47]. However, female transmission of the *hap13-1* heterozygous plants was comparable to that of wild type [27], suggesting that the female gametophytic defect of the homozygous *hap13-1* plants was sporophytic. Indeed, only in the homozygous *hap13-1* mutant did we observed defective FG, consistent with the result obtained by using the egg cell-specific marker *Pro*_*DD45*_:GUS (S1 Fig). Indeed, it’s known that gametophytic development relies on surrounding sporophytic tissues [3,7,48,49]. Mutations at genes controlling integument growth, such as *SUB*, often interfered with the formation of embryo sacs [14]. Our results seem to suggest that outer integuments play a key role in the sporophytic controlled female gametogenesis because manipulation of vesicle trafficking routes by *Pro*_*INO*_:HAP13-RNAi was sufficient to disrupt the patterning of FGs, suggesting a non-cell-autonomous mechanism. Interestingly, in this case, inner integuments were morphologically intact. How signals from outer integuments transmit through inner integuments to instruct the development of FG is unknown. In addition, inner integuments were suggested sufficient for female gametogenesis [50]. Whether specific signaling pathways are involved in the communication between two generations in *hap13-1* will certainly be a topic for future investigations.

## Materials and Methods

### Plant materials and growth conditions

Arabidopsis mutants and transgenic lines, including *hap13-1* [27], SUBg-YFP [35], PIN1::GFP [8], and DR5:GFP [40] were described. Arabidopsis Ws ecotype was used as the wild type. Arabidopsis plants were grown as described [51]. Stable transgenic plants were selected on half-strength MS supplemented with 30 μg/ml Basta salts, 25 μg/ml Hygromycin B, or 50 μg/ml Kanamycin.

### RNA-extraction and real time quantitative PCR

Total RNAs for detecting *HAP13* transcripts in *Pro*_*INO*_:HAP13-RNAi lines were extracted from mature unfertilized ovules using a Qiagen RNeasy plant mini kit according to manufacture’s instructions. Oligo(dT)-primed cDNAs were synthesized using Superscript III reverse transcriptase with on-column DNase II digestion (Invitrogen). Real time quantitative PCRs were performed as described [51]. Primers are listed in S1 Table.

### Plasmid construction

All constructs were generated using the Gateway technology (Invitrogen) except the RNAi construct. Entry vectors were generated using pENTR/D/TOPO (Invitrogen). Coding sequences or promoter sequences were cloned with the following primer pairs: ZP1781/ZP1782 for *Pro*_*INO*_ (2012 bp before the start codon of *INO*); ZP96/ZP97 for *Pro*_*DD45*_ (1003 bp before the start codon of *DD45*). The destination vector used to generate *Pro*_*DD45*_:GUS and *Pro*_*INO*_:GUS were described previously [52]. Expression vectors were obtained by combining respective entry vectors and destination vectors in LR reactions using LR Clonase II (Invitrogen). HAP13-RNAi fragment (401 bp to 700 bp of its coding sequence) was amplified with the primer pair ZP1650/ZP1651. The resultant PCR products were sub-cloned into the RNAi vector pTCK303 [53] to obtain the *Pro*_*UBQ10*_:HAP13-RNAi construct. Later, *Pro*_*UBQ10*_ was replaced with *Pro*_*INO*_ to generate *Pro*_*INO*_:HAP13-RNAi. A *Pro*_*INO*_:NLS-YFP expression cassette was amplified with primers ZP3872/ZP3873 containing the restriction enzyme site *Sac*I and *Eco*RI, respectively. The PCR fragment was inserted into the expression vector to generate the *Pro*_*INO*_:HAP13-RNAi *Pro*_*INO*_:NLS-YFP construct. Primers are listed in S1 Table.

### Phenotype and histochemical analyses

Histochemical GUS staining, ovule whole-mount clearing, semi-thin sections, and SEMs were performed as described [51]. Aniline blue staining of pollen tubes growing inside pistils was performed as described [54].

### Immunofluorescence labeling and fluorescence microscopy

Whole-mount immuno-fluorescence staining of ovules using an anti-LURE antibody [29] was performed as described [55]. Briefly, unfertilized mature pistils were placed in a fixative solution (3.7% paraformaldehyde, 1 mM CaCl_2_, 1 mM MgSO_4_, 50 mM HEPES (pH 7.4), 5% sucrose, pH8.0 with NaOH) for 10 min with vacuum desiccation at room temperature (RT). The fixative solution was then removed and pistils were washed three times for 10 min each with the PME buffer (50 mM PIPES, 1 mM MgCl_2_, 5 mM EGTA, pH6.8 with NaOH). The fixed pistils were treated further for permeation sequentially with a PME buffer, cellulose solution, and 3% IGEPAL CA-630. After permeation, pistils were sequentially incubated with the primary antibody (anti-LURE antibody, 1:300) and second antibody (FITC-labeled goat anti mouse, 1:100), followed by tissue mounting and CLSM examination. Uptake of FM4-64, BFA treatment and CLSM were performed as described [27]. Optical sectioning of ovules was performed as described [7,56].

### Accession numbers

Sequence data from this article can be found in the GenBank databases under the following accession numbers: At1g60780 for *HAP13*, At2g21750 for *DD45*, At1g23420 for *INO*, At5g43510 for *LURE1*.*2*.

## Supporting Information

S1 Fig Defective embryo sac formation of *hap13-1* is sporophytic. (A-C) Histochemical analysis of wild-type (A), heterozygous *hap13-1* (B), or homozygous *hap13-1* (C) pistils transformed with *Pro*_*DD45*_:GUS. Percentage of ovules showing GUS signals is shown on top of each image. Results are means ± standard error (s.e.m). In total, 18 pistils were analyzed for each genotype. Bars = 200 μm.
(TIF)

S2 Fig Protein but not transcript abundance of *SUB* is reduced in *hap13-1*. (A-B) CLSM of a representative stage 2-III ovule from SUBg-YFP in wild type (A) or in *hap13-1* (B) with the same illumination. The output images have not been brightened. Bars = 10 μm. (C) Relative transcript abundance of *SUB* by quantitative real-time PCRs. Results shown are means ± s.e.m. Three biological replicates were analyzed. No significant difference was observed (One-way ANOVA, Tukey-Kramer test, P>0.05).
(TIF)

S3 Fig Recycling of SUB to the plasma membrane requires *HAP13*. CLSM of SUBg-YFP in roots of 4 DAG wild-type or *hap13-1* seedlings. Con, controls; WO, washout. Arrows point at BFA compartments. Bars = 10 μm.
(TIF)

S4 Fig Dynamic targeting of PIN1 is independent of *HAP13*. (A-F) CLSM of PIN1:GFP in roots of 4 DAG wild-type (A, C, E) or *hap13-1* seedlings (B, D, F) upon 1 min FM4-64 uptake (A, B), upon BFA treatment for 50 min (C, D), or after BFA washout (E, F). Bars = 25 μm.
(TIF)

S5 Fig Verification of *HAP13* downregulation by *Pro*_*UBQ10*_:HAP13-RNAi. (A-D) CLSM of 4 DAG roots of wild type (A, C) or of *Pro*_*UBQ10*_:HAP13-RNAi transgenics (B, D) upon 1 min FM4-64 uptake followed by BFA treatment for 50 min (A, B) or after BFA washout (C, D). BFA washout in wild type (WT BFA wo) leads to tonoplast targeting of FM4-64 as seen by reduced signals at BFA compartments while in *Pro*_*UBQ10*_:HAP13-RNAi transgenics (*HAP13-RNAi* BFA wo) FM4-64 is still largely trapped in BFA compartments. (E) Relative transcript abundance of *HAP13* in wild type or six *Pro*_*UBQ10*_:HAP13-RNAi transgenic lines by quantitative real-time PCRs. RNAs were extracted from 4 DAG seedlings. Results shown are means ± s.e.m. (N = 3). Means with different letters are significantly different (One way ANOVA, Tukey-Kramer test, P<0.05). Bars = 10 μm.
(TIF)

S6 Fig Histochemical analysis of *Pro*_*INO*_:GUS during ovule development. (A-F) Transverse sections of *Pro*_*INO*_:GUS pistils at various developmental stages. Arrowheads point at outer integuments. Bars = 20 μm for (A-C); 10 μm for (D-F).
(TIF)

S7 Fig The specific expression of *Pro*_*INO*_ is not compromised by *Pro*_*INO*_:HAP13-RNAi. Representative severely (A) or mildly (B) affected ovules from the *Pro*_*INO*_:HAP13-RNAi;*Pro*_*INO*_:NLS-YFP transgenic plants. Shaded areas are either outer integuments (oi) or inner integuments (ii). The bright-field and YFP channel overlays are side-by-side with their corresponding YFP channel images. Hardly any signal was detected in the inner integuments. Bars = 20 μm.
(TIF)

S1 Table Primers used in this work. (DOC)
