# Supplementary figures and images for: HAPLESS13-Mediated Trafficking of STRUBBELIG Is Critical for Ovule Development in Arabidopsis
Source: PLoS Genet. 2016 Aug 19;12(8):e1006269. doi: 10.1371/journal.pgen.1006269 (PMC4991792; doi:10.1371/journal.pgen.1006269)

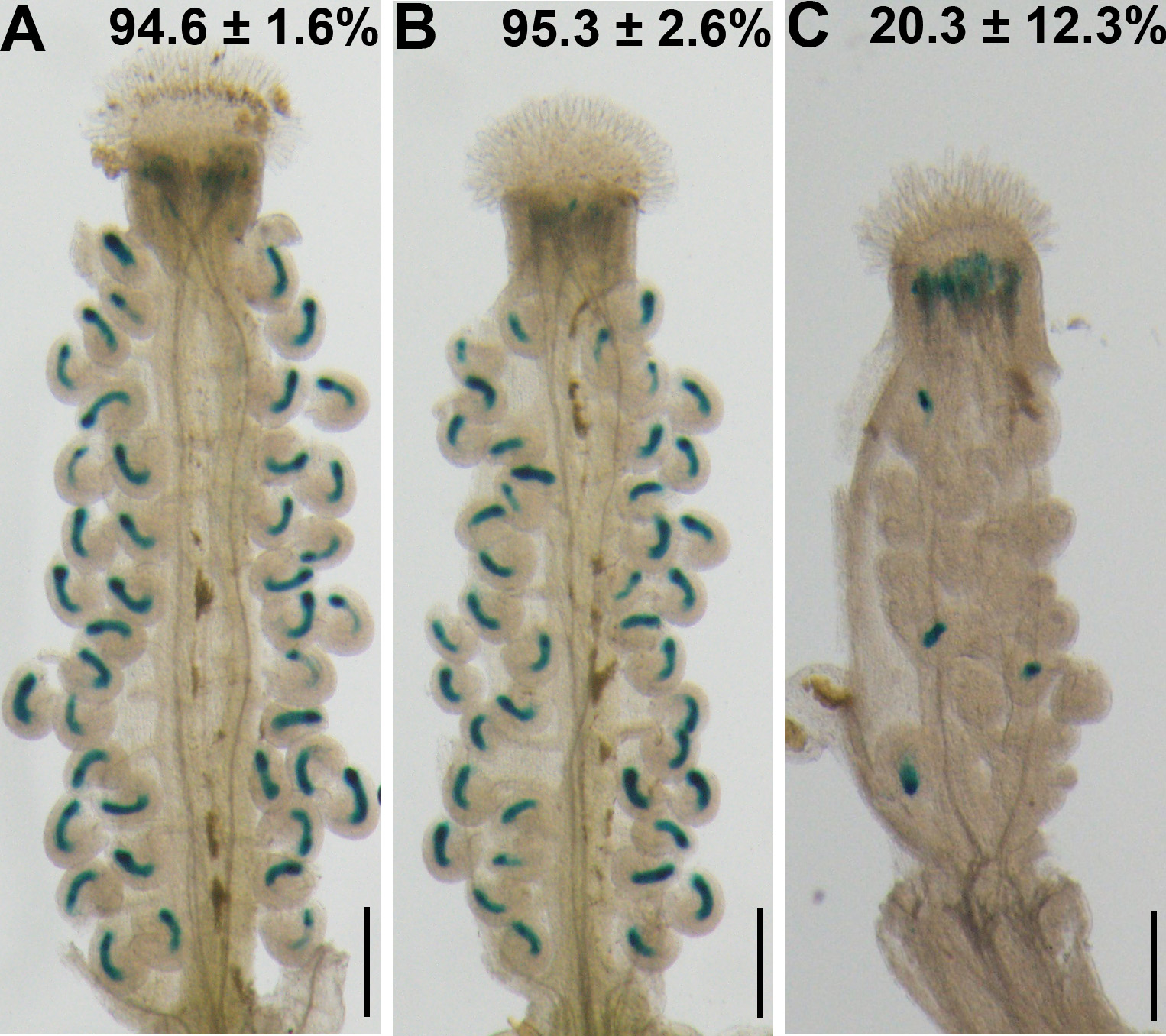

Supplement: S1 Fig — (A-C) Histochemical analysis of wild-type (A), heterozygous hap13-1 (B), or homozygous hap13-1 (C) pistils transformed with ProDD45:GUS. Percentage of ovules showing GUS signals is shown on top of each image. Results are means ± standard error (s.e.m). In total, 18 pistils were analyzed for each genotype. Bars = 200 μm. (TIF) [file pgen.1006269.s001.tif]

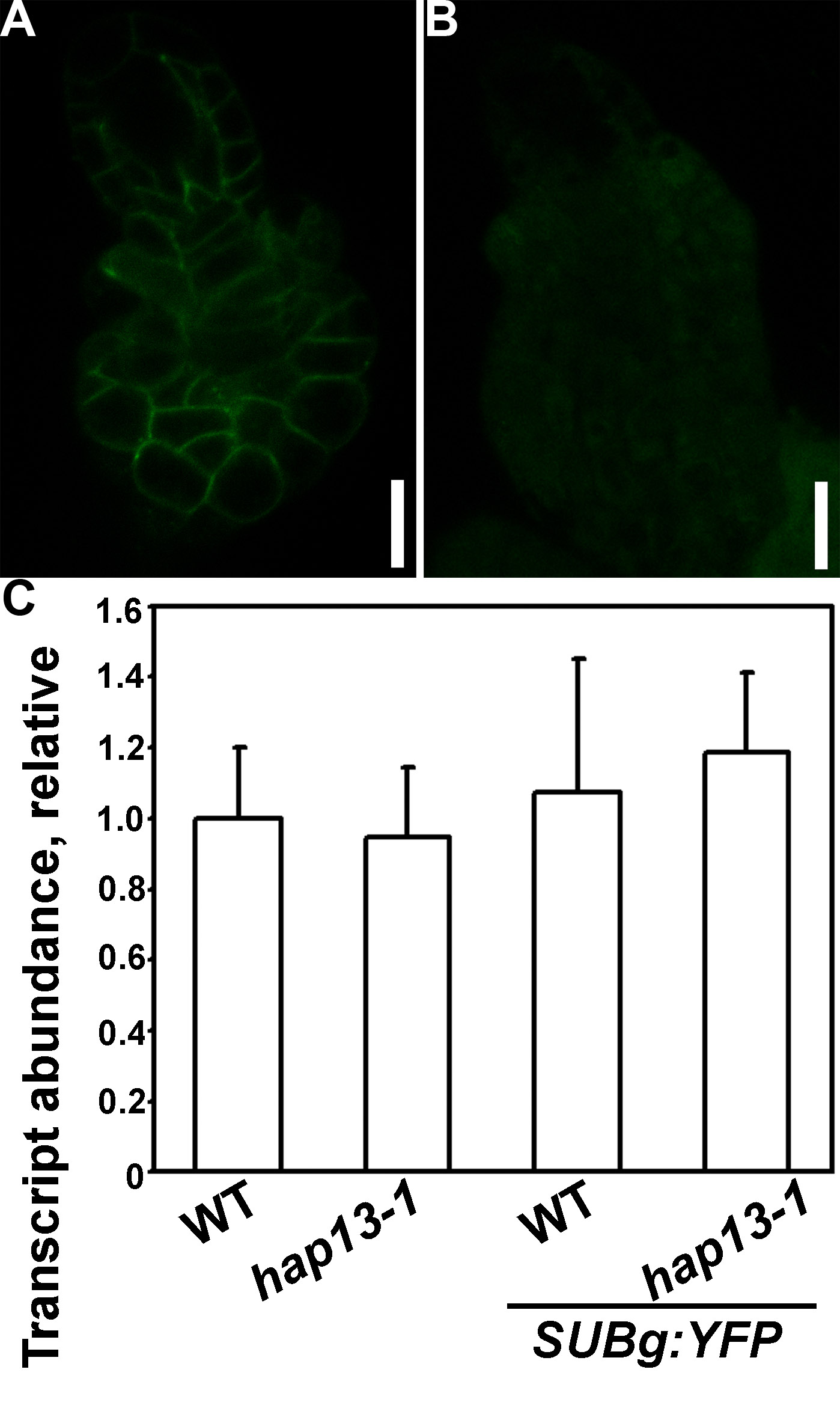

Supplement: S2 Fig — (A-B) CLSM of a representative stage 2-III ovule from SUBg-YFP in wild type (A) or in hap13-1 (B) with the same illumination. The output images have not been brightened. Bars = 10 μm. (C) Relative transcript abundance of SUB by quantitative real-time PCRs. Results shown are means ± s.e.m. Three biological replicates were analyzed. No significant difference was observed (One-way ANOVA, Tukey-Kramer test, P>0.05). (TIF) [file pgen.1006269.s002.tif]

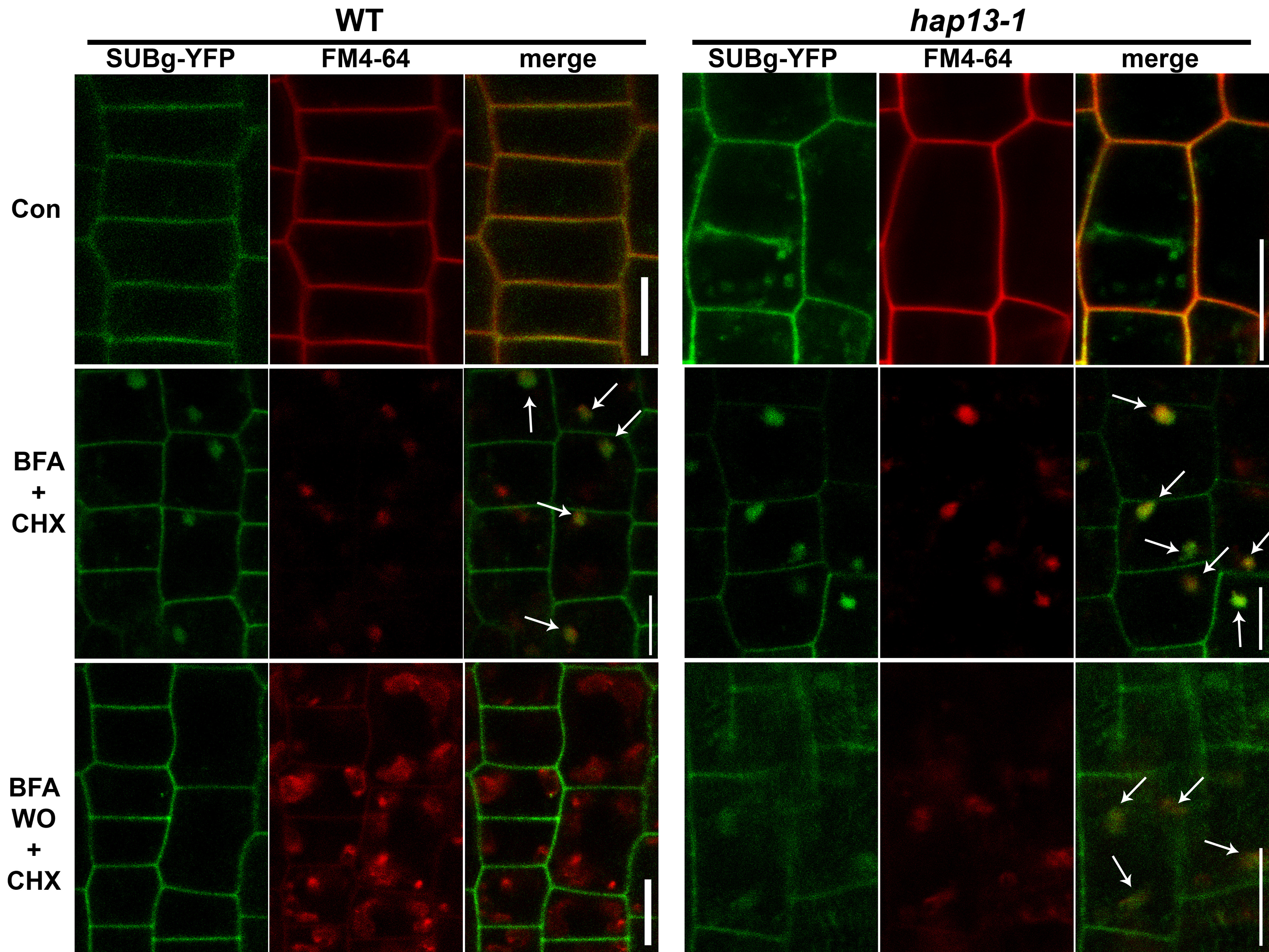

Supplement: S3 Fig — CLSM of SUBg-YFP in roots of 4 DAG wild-type or hap13-1 seedlings. Con, controls; WO, washout. Arrows point at BFA compartments. Bars = 10 μm. (TIF) [file pgen.1006269.s003.tif]

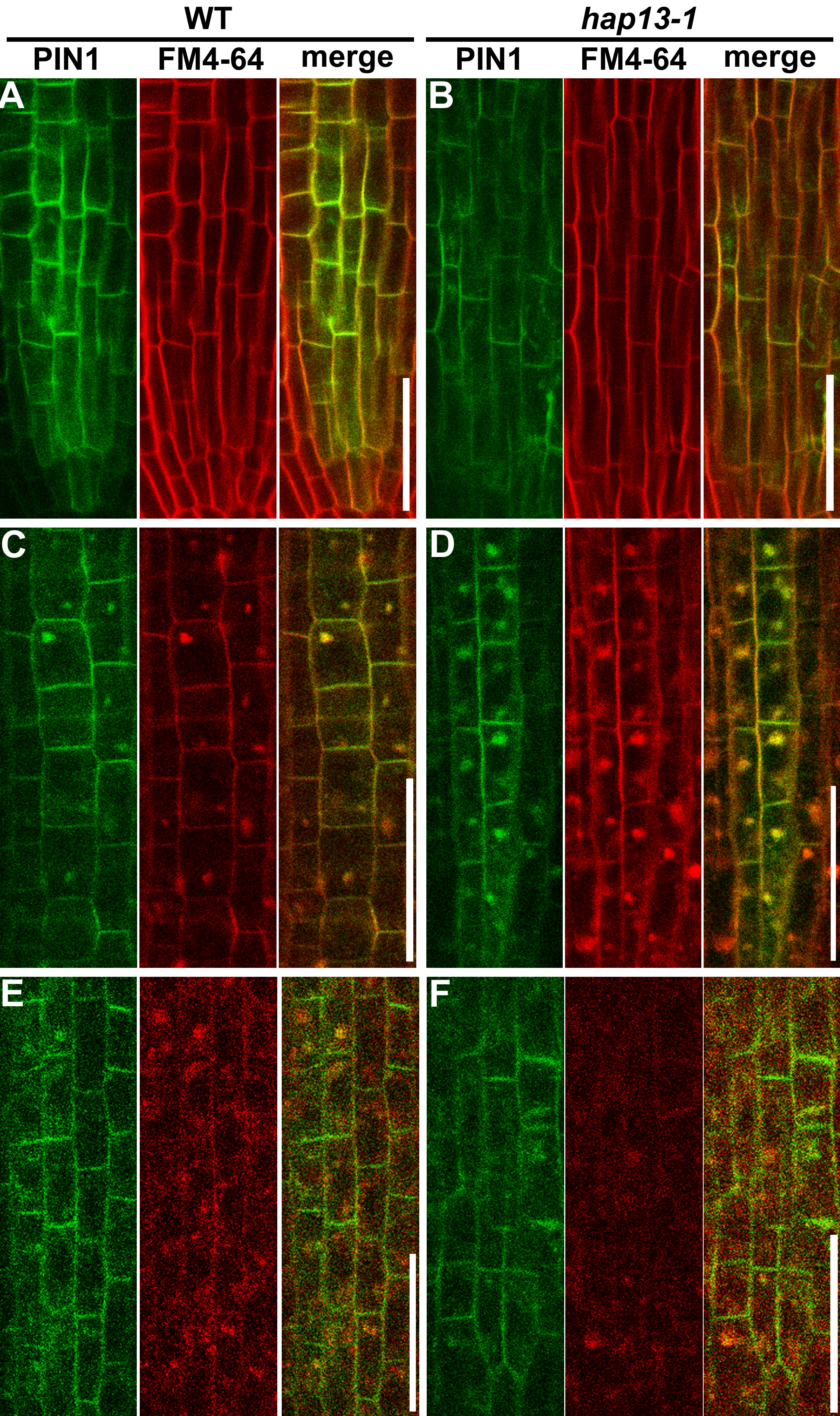

Supplement: S4 Fig — (A-F) CLSM of PIN1:GFP in roots of 4 DAG wild-type (A, C, E) or hap13-1 seedlings (B, D, F) upon 1 min FM4-64 uptake (A, B), upon BFA treatment for 50 min (C, D), or after BFA washout (E, F). Bars = 25 μm. (TIF) [file pgen.1006269.s004.tif]

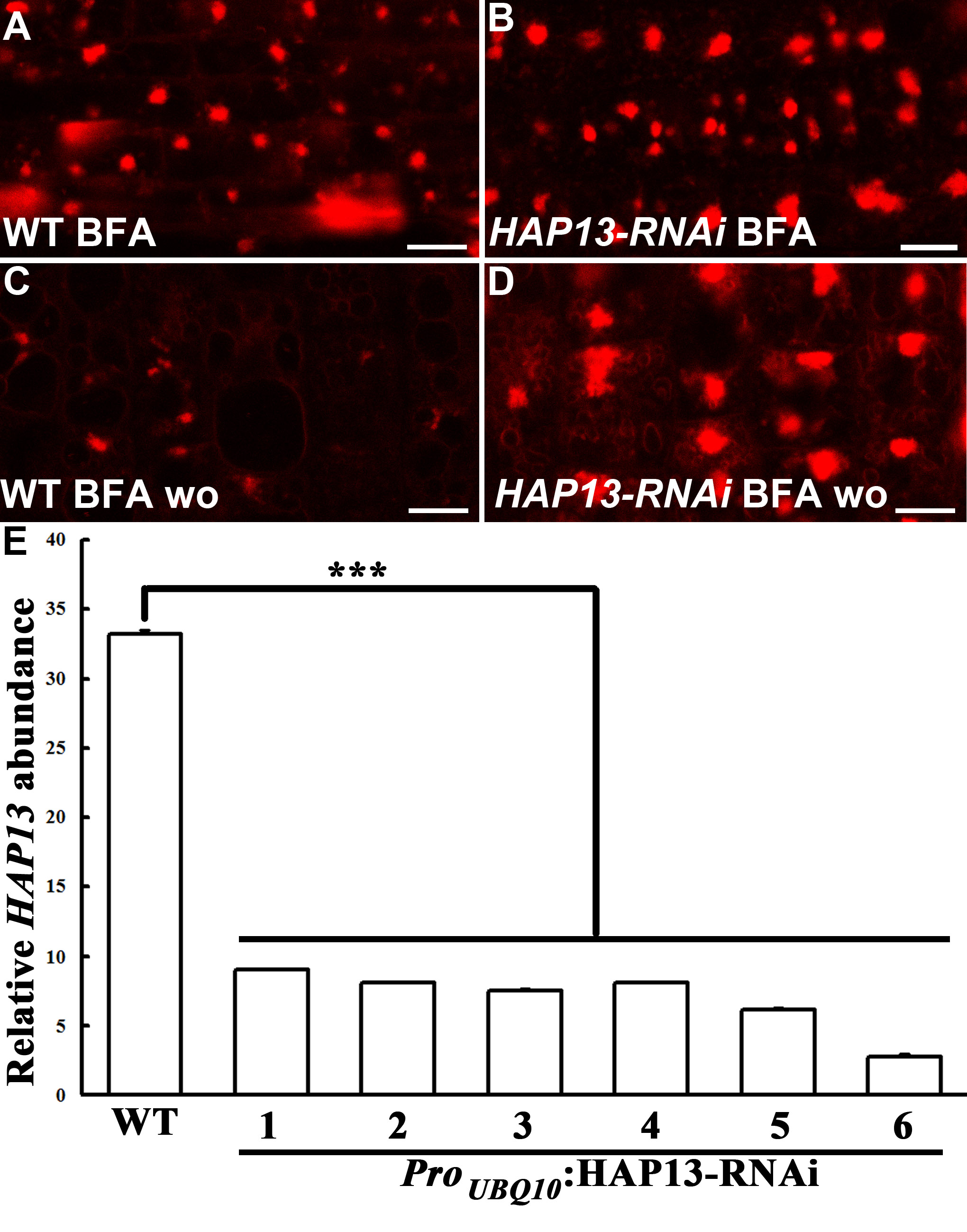

Supplement: S5 Fig — (A-D) CLSM of 4 DAG roots of wild type (A, C) or of ProUBQ10:HAP13-RNAi transgenics (B, D) upon 1 min FM4-64 uptake followed by BFA treatment for 50 min (A, B) or after BFA washout (C, D). BFA washout in wild type (WT BFA wo) leads to tonoplast targeting of FM4-64 as seen by reduced signals at BFA compartments while in ProUBQ10:HAP13-RNAi transgenics (HAP13-RNAi BFA wo) FM4-64 is still largely trapped in BFA compartments. (E) Relative transcript abundance of HAP13 in wild type or six ProUBQ10:HAP13-RNAi transgenic lines by quantitative real-time PCRs. RNAs were extracted from 4 DAG seedlings. Results shown are means ± s.e.m. (N = 3). Means with different letters are significantly different (One way ANOVA, Tukey-Kramer test, P<0.05). Bars = 10 μm. (TIF) [file pgen.1006269.s005.tif]

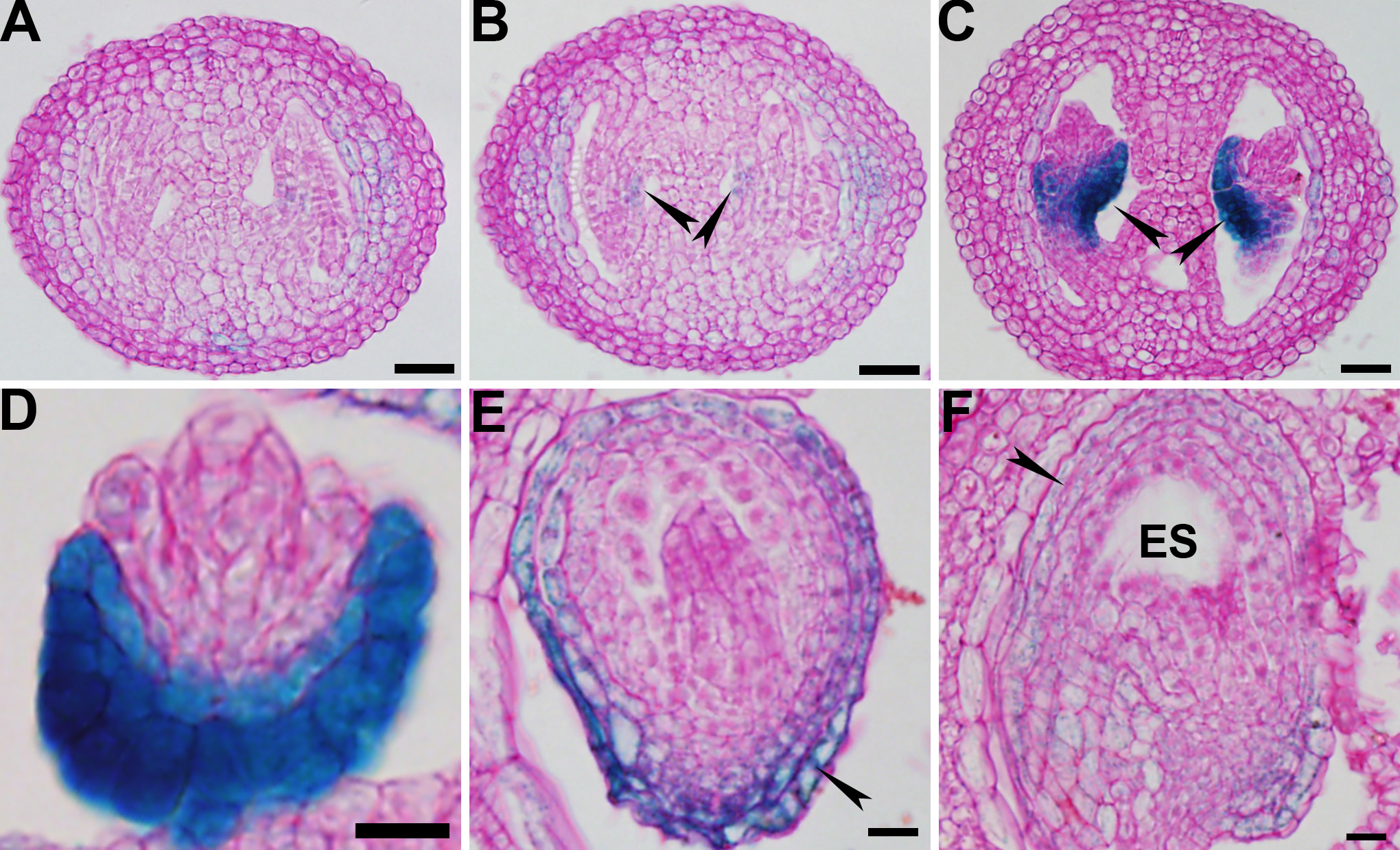

Supplement: S6 Fig — (A-F) Transverse sections of ProINO:GUS pistils at various developmental stages. Arrowheads point at outer integuments. Bars = 20 μm for (A-C); 10 μm for (D-F). (TIF) [file pgen.1006269.s006.tif]

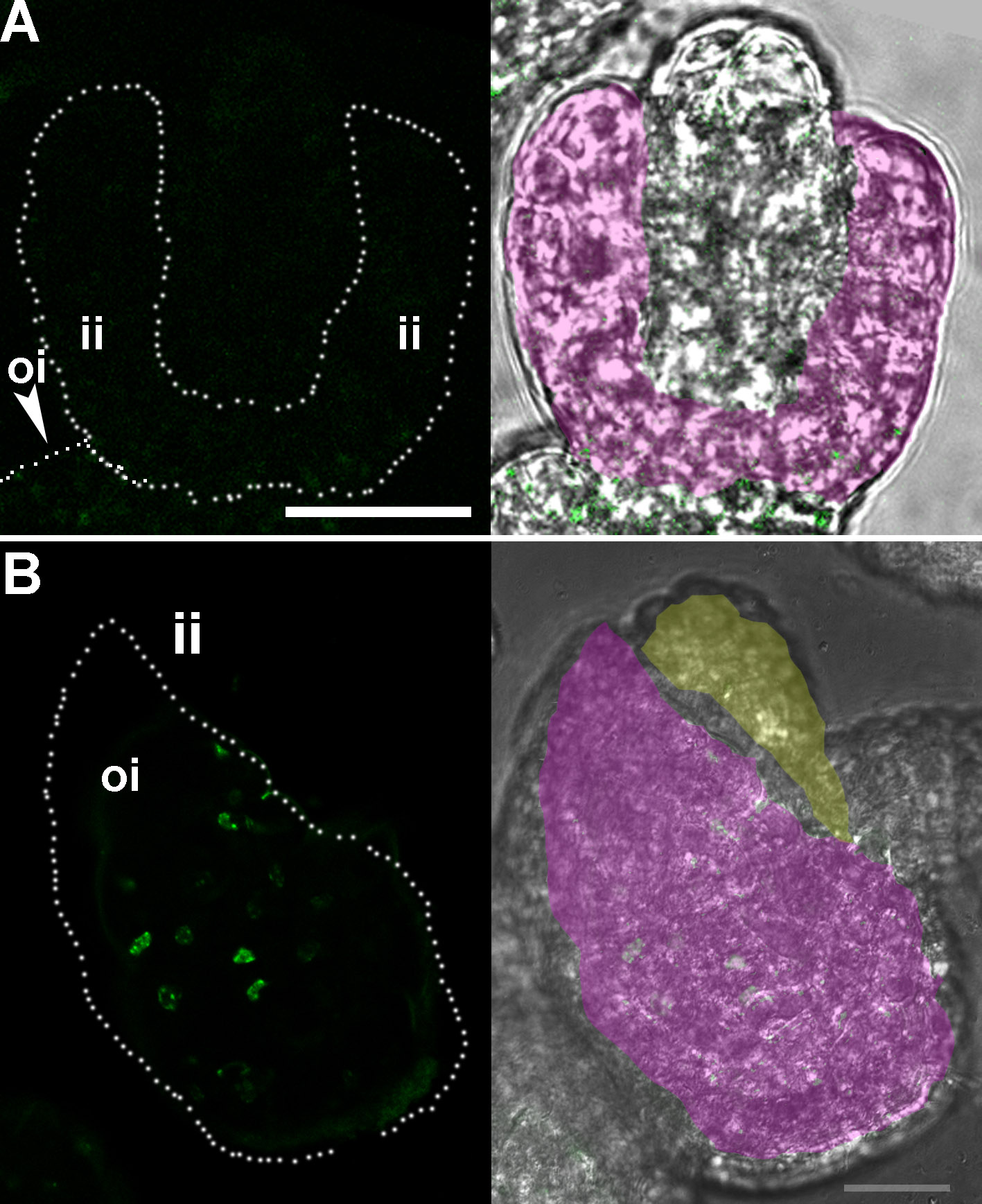

Supplement: S7 Fig — Representative severely (A) or mildly (B) affected ovules from the ProINO:HAP13-RNAi;ProINO:NLS-YFP transgenic plants. Shaded areas are either outer integuments (oi) or inner integuments (ii). The bright-field and YFP channel overlays are side-by-side with their corresponding YFP channel images. Hardly any signal was detected in the inner integuments. Bars = 20 μm. (TIF) [file pgen.1006269.s007.tif]
