# Supplementary material for: HAPLESS13-Mediated Trafficking of STRUBBELIG Is Critical for Ovule Development in Arabidopsis
Source: PLoS Genet. 2016 Aug 19;12(8):e1006269. doi: 10.1371/journal.pgen.1006269 (PMC4991792; doi:10.1371/journal.pgen.1006269)
Supplement: S1 Table — (DOC) [file pgen.1006269.s008.doc]

**S1** Table. Primers used in this work.

| Application | | No. | 5’-3’ sequences |
| --- | --- | --- | --- |
| qPCRs | *HAP13* | ZP3141 | GATGCATATAGAATGGAAGTTACAC |
|  |  | ZP3142 | ACAACATCAGACCTGACAATTTGC |
| cloning | *Pro_INO_* | ZP1781 | CACCGTTATGTCTTTTTGCTTTTGGAATT |
|  |  | ZP1782 | AGAGAGTGTGTGTGTACGATGAAT |
|  |  | ZP1703 | ATAAGCTTGTTATGTCTTTTTGCTTTTGGAATT |
|  |  | ZP1704 | ATGGATCCAGAGAGTGTGTGTGTACGATGAAT |
|  |  | ZP1472 | ATATGGTACCGTTATGTCTTTTTGCTTTTGGAATT |
|  |  | ZP1473 | ATATGACGTCAGAGAGTGTGTGTGTACGATGAAT |
|  | *Pro_DD45_* | ZP96 | CACCTAAATGTTCCTCGCTGACGTA |
|  |  | ZP97 | TATTCTTTCTTTTTGGGGTTTTTG |
|  | HAP13-RNAi | ZP1650 | ATATGGATCCGAGCTCTCAGTGAATTCATCAAGACTGAT |
|  |  | ZP1651 | ATATGGTACCACTAGTTGGCTTTTCCCTTAGTTGC |
|  | *Pro_INO_:NLS-YFP* | ZP3872 | ATATGAGCTCGTTATGTCTTTTTGCTTTTGGAATTTAG |
|  |  | ZP3873 | ATATGAATTCTCACTGGATTTTGGTTTTAGGAATTAG |
